# Epidemiological and clinical characteristics of patients in the alveolar echinococcosis registry, France, 1982 to 2021

**DOI:** 10.2807/1560-7917.ES.2025.30.32.2500041

**Published:** 2025-08-14

**Authors:** Jenny Knapp, Florent Demonmerot, Houssein Gbaguidi-Haore, Carine Richou, Dominique Angèle Vuitton, Anne-Pauline Bellanger, Solange Bresson-Hadni, Laurence Millon, on behalf of the FrancEchino network

**Affiliations:** 1 National Reference Centre for Echinococcoses, University Hospital of Besançon, Besançon, France; 2 UMR CNRS 6249 Chrono-environnement Laboratory, Marie and Louis Pasteur University, Besançon, France; 3 Laboratory of Hospital Hygiene, University Hospital of Besançon, France; 4 Department of Hepatology, University Hospital of Besançon, Besançon, France; 5 The members of the FrancEchino network are listed under Acknowledgements

**Keywords:** alveolar echinococcosis, *Echinococcus multilocularis*, follow-up, French patient registry, epidemiological data, clinical data

## Abstract

**BACKGROUND:**

*Echinococcus multilocularis* is a parasite causing alveolar echinococcosis (AE), a severe disease affecting primarily the liver. Surveillance of this non-notifiable disease in France is performed by the National Reference Center for Echinococcoses (NRC-E).

**AIM:**

We aimed to analyse changes in epidemiological, clinical and survival data of patients with AE over time.

**METHODS:**

We described and analysed data from 906 AE patients reported to the NRC-E over three periods: 1982–1999, 2000–2010 and 2011–2021, using regression methods and survival analysis methods.

**RESULTS:**

At diagnosis, the median age of the patients was 60.0 years, most (770; 85.0%) resided in an endemic region and 483 (53.3%) in a rural area. The percentage of asymptomatic patients increased significantly from 19.1% (48/251) in 1982–1999 to 56.2% (209/372) in 2011–2021 (p < 0.001). The number of patients with weakened immune systems increased significantly in 2000–2021 (p < 0.001). Most (761/836; 91.0%) patients were treated with antiparasitic drugs and 402 (44.4%) underwent surgery. The number of surgical interventions decreased significantly during the study period (p = 0.007). Palliative surgery decreased, curative hepatic resection became more commonly performed (p < 0.001). Multivariate analysis showed a lower risk of death in the first 10-year follow-up in patients diagnosed after 2000 and those receiving benzimidazoles (sub-distribution hazard ratio (SHR) = 0.43; 95% confidence interval (CI): 0.28–0.66; p < 0.001).

**CONCLUSION:**

International recommendations for treating all patients with benzimidazoles and favouring curative hepatic resection, whenever possible, were generally followed and associated with better survival. We recommend national and European-wide registries to ensure effective surveillance of AE.

**Key public health message**

**What did you want to address in this study and why?**

Alveolar echinococcosis (AE), a parasitic disease mainly transmitted by foxes, is a severe infection caused by *Echinococcus multilocularis.* The parasite affects primarily the liver. We aimed to describe and analyse changes over time in patients with AE in France, based on a national registry of data collected from 906 patients over 40 years between 1982 and 2021.

**What have we learnt from this study?**

Diagnoses of AE increased nearly 2.5-fold during the study period. Most (n = 770) AE patients were living in areas where the parasite is known to exist, but the number of patients living in other areas increased significantly. Most (761/836; 91.0%) patients were treated with antiparasitic drugs and 402 (44.4%) underwent surgery. The number of surgical interventions decreased significantly during the study period (p = 0.007).

**What are the implications of your findings for public health?**

We observed an increase in the number of patients with AE: in people without symptoms at diagnosis, among patients with weakened immune systems and people living outside of at-risk areas. Establishment of national and European-wide registries and involving physicians of different specialities is essential for surveillance of AE. A wide collection of data can help identify risk factors and formulate recommendations for prevention.

## Introduction

Alveolar echinococcosis (AE) is a parasitic disease caused by the larvae (metacestodes) of *Echinococcus multilocularis*. In humans, AE develops as tumour-like lesions, primarily in the liver but the parasite can affect other organs [1]. In immunocompetent people, the disease progresses slowly, often with symptoms appearing over 5–15 years after the exposure [2]. The parasite is found primarily in the northern hemisphere, with major endemic areas in western China [3]. From 1982 to 2000, 42% of the European AE patients were diagnosed in France [4]. The life cycle of the parasite involves a definitive host, usually the red fox (*Vulpes vulpes*), and an intermediate host, mainly rodents from the genera *Microtus* and *Arvicola* [5].

Diagnosis is usually made based on clinical and epidemiological findings. Imaging techniques ultrasound (US), computerised tomography (CT) and magnetic resonance imaging (MRI) are currently used [1]. Detection of specific antibodies against *E. multilocularis* can aid diagnosis but is not necessary for a diagnosis [6]. Confirmation requires the use of pathological or molecular methods but is not mandatory to assert the diagnosis [1]. Treatment decisions are based on the World Health Organization (WHO) Informal Working Group on Echinococcosis (IWGE) classification of AE: PNM: parasitic mass in the liver (P), involvement of neighbouring organs (N) and metastasis (M) [7], based on lesion extent [1]. Imaging techniques, such as 18F-labelled fluoro-2-deoxyglucose positron emission tomography combined with CT (18F-FDG PET/CT) and serological markers can be used to assess parasite viability [3].

Prior to 1985, partial surgical resection was the only treatment option. In the 1980s, WHO-supported multicentre studies indicated that high-dose benzimidazoles (albendazole (ABZ) and mebendazole (MBZ)) were effective, leading to their widespread use [1]. The WHO-IWGE guidelines recommend curative surgery combined with up to 2 years of ABZ therapy [1]. When AE lesions are inoperable, lifelong treatment with ABZ is needed [1]. However, these drugs are parasitostatic, not parasiticidal, slowing but not eliminating parasite progression. Side effects, such as hepatotoxicity, neutropenia and alopecia, require close monitoring and can lead to a therapeutic impasse [8]. However, in specific cases, based on the imaging results (18-FDG PET/CT and/or MRI) and favourable specific antibody kinetics, antiparasitic treatment can be discontinued after multidisciplinary consultation and under careful monitoring. Patients with severe symptoms and life-threatening illness can ultimately be treated with liver transplantation [9] or ex vivo resection with auto-transplantation [10].

Over the past 40 years, prevalence of *E. multilocularis* in red foxes has increased, particularly since the year 2000, with urban parasite cycles emerging and endemic areas expanding [5]. Risk factors for humans include living in endemic regions or engaging in activities that expose individuals to parasite eggs, such as gardening or hunting [5]. Recent studies from France, Germany and Switzerland highlight an increased incidence of AE in people with weakened immune systems, suggesting AE as an opportunistic infection [11-13].

We analysed data from AE patients registered in France from 1982 to 2021, focusing on three time periods to examine changes in diagnosis, clinical features and patient prognosis.

## Methods

### Data collection

We collected demographic, epidemiological and clinical data on patients diagnosed from 1982 to 2021. More details on the type of data collected are available in Supplementary Table 1. Data on patients diagnosed from 1982 to 1998 were retrospectively collected 1997–1998 within the framework of the EurEchinoReg registry [4], and data on patients diagnosed after 1999 were from the French national AE (FrancEchino) registry. FrancEchino was first formally appointed in 2003 to manage the registry. In 2012, the French National Public Health Agency formally appointed the National Reference Center for Echinococcoses (NRC-E) to sustain the French AE registry and to provide yearly epidemiological data to European Centre for Disease Prevention and Control (ECDC). From the very beginning, completeness of the data collected over the French territory was the goal of the EurEchinoReg and FrancEchino registries [4,14]. Cases were directly reported by physicians who requested a confirmation of diagnosis or treatment expertise from the NRC-E. Data on cases were also actively obtained through bi-annual systematic surveys from (i) clinical pathology laboratories of all French public hospitals; (ii) clinical microbiology laboratories of all French public hospitals and private clinical microbiology laboratories using serological tests on *E. multilocularis* and (iii) hospital pharmacies, which are in charge of dispensing ABZ and MBZ in France according to the French regulations. Every suspected case was then evaluated by the NRC-E and included in the database if the case was confirmed or probable [1].

We divided the dataset into three time periods, based on the evolution of medical imaging practices, the establishment of expert consensus recommendations (in 2001 and 2010) [1,15], the implementation of antiparasitic treatment according to these international guidelines, and the emergence of cases among patients with weakened immune systems [11]. The first period (period 1, P1) from January 1982 to December 1999 included the data collected within the EurEchinoReg study [4], period 2 (P2) was from January 2000 to December 2010 and period 3 (P3) from January 2011 to December 2021.

The terminology of echinococcosis used in this study was according to the international consensus report [16].

### Data analysis

All analyses were conducted on pseudonymised data using the software Stata version 14.1 (https://www.stata.com) and R version 4.4.1 (http://www.R-project.org). A p value < 0.05 was considered statistically significant.

#### Epidemiological data analysis

In the epidemiological analysis, we aimed to describe the temporal trends and the distribution of at-risk activities among AE patients.

We collected data on the place of residence of the patient (geographical location, urbanisation status) and on recreational (hunting, wild plant consumption, gardening) and occupational activities. The occupational activities were divided into socio-professional categories (SPC) in eight categories from the nomenclature of the French National Institute of Statistics and Economic Data (INSEE; https://www.insee.fr/fr/information/2497952) and occupational at-risk activities (OARA). The at-risk activities were further categorised into four groups based on the potential exposure to AE: (i) OARA-1: farmers (active or retired), shepherds, gardeners, vegetable producers; (ii) OARA-2: farm workers; (iii) OARA-3: forestry workers; (iv) OARA-4: other at-risk outdoor occupations for *E. multilocularis* exposure. These data are presented in Supplementary Methods S1. In France, the endemic area for *E. multilocularis* infection covers 22 French administrative areas (‘départements’), abbreviated as departments at risk (DARs) for AE, located in eastern and central France [17]. The proportion of AE patients living in the DARs was estimated for the three study periods. Additionally, history of travel to or residence in a DAR before AE diagnosis was recorded.

To assess temporal trends in the epidemiological characteristics of patients with AE, chi-square test and Kruskal-Wallis test were used.

#### Clinical data

Symptoms (Table 1), incidental discovery with a compatible image or serological test results and findings of abdominal ultrasound screening were recorded when the patient was diagnosed with AE. A patient may have presented no symptoms, one symptom or several symptoms. Information on weakened immune system (IS) of the patient was recorded as previously described [11]. Since 2002, characterisation of AE lesions has included the PNM stage according to the WHO-IWGE classification system [1,7]. Information on antiparasitic and surgical treatment, if any, was recorded.

**Table 1:** Reported symptoms among patients with alveolar echinococcosis at diagnosis, France, 1982–2021 (n = 312)^a,b^

Symptoms	Number of reports	%
Abdominal pain	176	56.4
Abscess	11	3.5
Cholangitis	15	4.8
Cholestatic jaundice	85	27.2
Hepatomegaly	65	20.8
Portal hypertension	5	1.6
Pruritus	52	16.7
Other symptoms	68	21.8
One symptom	157	50.3
Multiple symptoms	155	49.7

^a^ A patient could have more than one symptom.

^b^ Of the 453 symptomatic patients, data were missing from 141 patients.

To assess temporal trends in the clinical characteristics of patients with AE, chi-square tests and Fisher’s f-test were run using a linear regression analysis.

#### Survival data and survival probability

Survival data were obtained from: (i) physicians of the FrancEchino network for patient follow-up; (ii) the CépiDc INSERM Laboratory (French Epidemiological Center on Medical Causes of Death; https://www.cepidc.inserm.fr ) and (iii) INSEE (https://www.insee.fr/en/accueil). The vital status (alive, dead, lost to follow-up) by 31 December 2021 was used as an endpoint. The age at death of AE patients and the life expectancy in the French population from 1982 to 2021 were compared.

The vital status of the patients during 10-year follow-up was analysed and compared between patients with an AE diagnosis before or after 2000. Details on these comparisons are presented in Supplementary Methods S2. Survival patterns were determined and compared using Kaplan-Meier survival curves and log-rank tests. A competing-risks regression model was then constructed with 10-year all-cause death as the event of interest and loss to follow-up as the competing event, presented in Supplementary Methods S2.

## Results

### Analysis of epidemiological data

#### Demographic data

**Figure 1:**
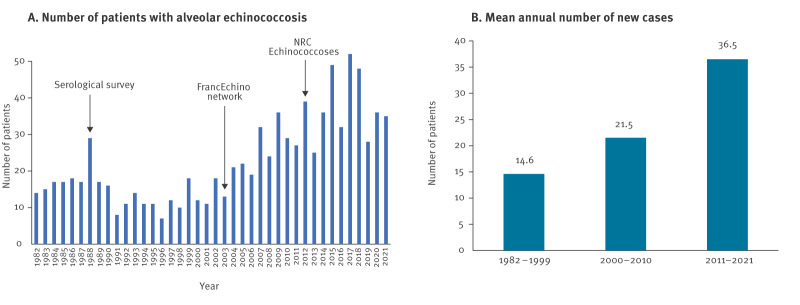
Number of patients with alveolar echinococcosis and mean annual number of new patients with alveolar echinococcosis, by year and surveillance activity, France, 1982–2021 (n = 906)

A total of 906 patients received an AE diagnosis from 4 January 1982 to 18 November 2021 (Figure 1A) and were registered in the database from 1 January 1982 to 31 December 2021. Of these, 262 patients were diagnosed in 1982–1999, 237 patients in 2000–2010 and 407 patients in 2011–2021. The mean annual number of new diagnoses during these periods were 14.6, 21.5 and 36.5, respectively (Figure 1B), and the mean annual incidences per 100,000 inhabitants were 0.026, 0.035 and 0.061, respectively. The sex ratio was 1.068 during the study period. The median age at diagnosis was 58, 60 and 63 years, respectively (range: 10–91 years). The mean age at diagnosis increased significantly over time (p = 0.004): 1982–1999: 56.3 years (95% confidence interval (CI): 54.6–58.1), 2000–2010: 58.8 years (95% CI: 56.6–61.0) and 2011–2021: 59.9 years (95% CI: 58.4–61.5), and between 1982–1999 and 2011–2021 (p = 0.003).

NRC: National Reference Centre.

Panel A. Patients were diagnosed 4 January 1982–18 November 2021. The arrows mark specific surveillance activities (serological survey, establishment of the FrancEchino network and the NRC for Echinococcoses).

#### Place of residence, geographical distribution of patients with alveolar echinococcosis

**Figure 2:**
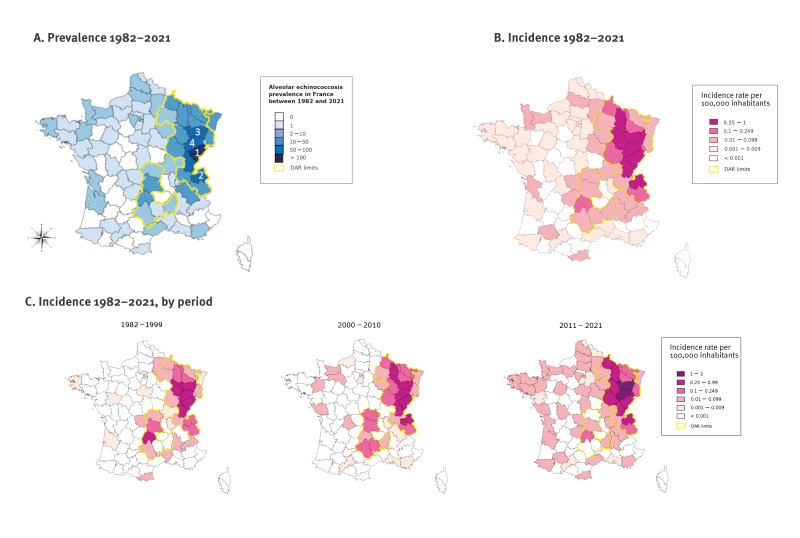
Geographical distribution of alveolar echinococcosis patients, by residence at diagnosis, France, 1982–2021 (n = 906)

Patients with AE were residing in 72 (75.8%) of the 95 departments of Metropolitan France (1982–1999: 35; 2000–2010: 38; 2011–2021: 62). The departments with the highest case numbers were Doubs (n = 156), Haute-Savoie (n = 100), Vosges (n = 92) and Haute-Saône (n = 69) (Figure 2A). In 2000–2010, Doubs and Vosges had the highest incidence, with 1–2 cases per year and per 100,000 inhabitants (Figure 2B). However, over time, patients were diagnosed throughout the French territory (Figure 2C).

DAR: department at risk.

Panel A. 1: Doubs; 2: Haute-Savoie; 3: Vosges; 4: Haute-Saône.

Most patients with AE (770/906; 85.0%) lived in the DARs. The proportion of patients diagnosed with AE in other departments than DARs increased significantly from 7.6% in 1982–1999 to 24.1% in 2011–2021 (p < 0.001). Of the 136 patients living outside the endemic areas, 51 reported that they made trips to or stayed in DARs, but 31 had not stayed in a DAR. Data from 52 patients were missing. Patients’ residences at diagnosis were in rural areas (n = 483; 53.3%), city centres (n = 206; 22.7%), suburbs (n = 157; 17.3%) and isolated cities (urban unit constituted by a single municipality) (n = 60; 6.6%), with an increase in suburbs and city centres over time. More details can be found in Supplementary Figure S1.

#### Occupational activities

The socio-professional activity (SPC) was documented for 740 (81.7%) patients. A total of 199 (26.9%) patients were active or retired farmers (SPC-1), the proportion of farmers with AE decreased significantly during the study period, but the proportion of executive and intellectual professionals as the proportion of employees increased (p < 0.001), as presented in Supplementary Figure S2. Occupational at-risk activities (OARA) were noted for 235 patients: 83.4% (196/235) were OARA-1, 5.1% (12/235) OARA-2, 6.8% (16/235) OARA-3 and 4.7% (11/235) were OARA-4.

#### Recreational activities

In total, 567 (62.6%) patients provided information about at-risk recreational activities of *E. multilocularis* exposure and indicated at least one at-risk item, as presented in Supplementary Figure S3. Of these patients, 467 (82.4%) picked berries and wild plants, 437 (77.1%) were gardening, 216 (38.1%) had contact with foxes, and 84 (14.8%) were hunting, 435 (76.7%) owned pets (dogs alone: 130 (22.9%), cats alone: 89 (15.7%), both animals: 188 (33.2%), without precision about the pet species: 28 (4.9%)). The association between berry picking, gardening and pet ownership was observed in 297 (52.4%) patients.

### Analysis of clinical data

#### Symptoms of patients with alveolar echinococcosis

We received information on symptoms of 846 (93.4%) patients. Over the study period, 453 (53.5%) patients were symptomatic and 393 (46.5%) were asymptomatic. Of the asymptomatic patients, 34 were diagnosed via a screening (13 of these in a screening of farmers in Doubs 1986–1989 [18] and 21 in an investigation of a patient’s family members). Thirty-nine patients had a family member with AE. The percentage of asymptomatic patients increased significantly from 19.1% (n = 48) in 1982–1999 to 56.2% (n = 209) in 2011–2021 (p < 0.001). Of the symptomatic patients with reported symptoms (n=312), 155 (49.7%) presented several symptoms or signs: 176 (56.4%) had abdominal pain, 65 (20.8%) hepatomegaly and 85 (27.2%) cholestatic jaundice (Table 1).

#### Patients with weakened immune systems at diagnosis

Information on a weakened immune system was documented for 155 (17.1%) of 906 patients: eight (3.1%) in 1982–1999, 45 (19.0%) in 2000–2010 and 102 (25.1%) in 2011–2021. The number of patients with weakened immune systems increased significantly in 2000–2021 (p < 0.001), as presented in Supplementary Figure S5. Of these 155 patients, 52 had cancer, 73 had cancer and another condition weakening the immune system, 43 had chronic or autoimmune inflammatory diseases, 19 had haematological malignancies, 11 had solid organ transplants, three had an HIV infection and 16 had other conditions (e.g. diabetes, corticosteroid therapy, cirrhosis).

#### Clinical classification of patients with alveolar echinococcosis

The PNM classification at diagnosis (published in 2006 [7] and used in our centre since 2002) was available for 553 patients, diagnosed in 2000–2021 (Table 2). More details are presented in Supplementary Table S2. Liver was the primary infection site in 541 (97.8%) patients: 191 (97%) 2000–2010 and 350 (98.3%) 2011–2021. Primary extrahepatic AE was observed in 12 (2.2%) patients. Liver AE involvement alone (P1–4N0M0) was recorded in 398 (72.0%) patients, liver and neighbouring organ involvement (P1–4N1M0) in 70 (12.7%), and liver and distant metastases without neighbouring organ involvement (P1–4N0M1) in 26 (4.7%) patients over the entire period. There were no significant differences in PNM stage over time.

**Table 2:** Scoring of the clinical status of patients with alveolar echinococcosis, France, 2000–2021 (n = 553)^a^

Stage	PNM	2000–2021 (n = 553)		2000–2010 (n = 197)		2011–2021 (n = 356)	
		n	%	n	%	n	%
Stages I–IIIa	P1–3N0M0	350	63.3	136	69.0	214	60.1
Stage IIIb	P4N0M0	48	8.7	7	3.6	41	11.5
	P1–3N1M0	49	8.9	22	11.2	27	7.6
Stage IV	P4N1M0	21	3.8	5	2.5	16	4.5
	P1–4N0–1M1	47	8.5	17	8.6	30	8.4
	P0N0–1M0–1	12	2.2	6	3.0	6	1.4
Other	Other PNM	26	4.7	4	2.0	22	6.2

M: metastasis; M0: no metastasis; M1: metastasis present; N: extra-hepatic involvement of neighbouring organs; N0: no regional involvement; N1: regional involvement of contiguous organs or tissues; NA: not applicable; PNM: categorisation of lesions; P: hepatic localisation of the parasite;P0: no detectable liver lesion; P1: peripheral lesions without proximal vascular and/or biliary involvement; P2: central lesions with proximal vascular and/or biliary involvement of one lobe; P3: central lesions with hilar vascular or biliary involvement of both lobes and/or with involvement of two hepatic veins; P4: any liver lesion with extension along the vessels and the biliary tree; WHO: World Health Organization informal working group on echinococcosis.

Stages I–IIIa: liver; stage IIIb: liver with or without neighbouring organ; stage IV: liver with neighbouring organ and/or metastasis.

^a^ Scoring was conducted according to the WHO-IWGE classification system [1,7].

#### Antiparasitic treatment

Antiparasitic therapy was initiated at diagnosis in 761 (91.0%) of 836 patients documented, with information about the antiparasitic molecule type for 750 patients. Albendazole was given to 90.8% (681/750), MBZ to 7.5% (56/750; 54 patients treated in 1982–1999), flubendazole to 0.7% (5/750, only in 1982–1999) and other antiparasitic drugs to 1.1% (8/750, only in 1982–1999). Albendazole accounted for 67.5% (139/206) of the antiparasitic treatments in 1982–1999, 99.5% (210/211) in 2000–2010, and 99.7% (332/333) in 2011–2021. Of these 836 patients, 464 (55.5%) were treated with antiparasitic drugs without surgery (1982–1999: 105/255 (41.2%); 2000–2010: 129/229 (56.3%); 2011–2021: 230/352 (65.3%)).

#### Surgical interventions

Of the 906 patients, 402 (44.4%) underwent surgery. The proportion of patients treated with surgery decreased significantly during the study period (1982–1999: 161 (61.5%); 2000–2010: 118 (49.8%) and 2011–2021: 123 (30.2%); p = 0.007). More information is presented in Supplementary Table S3. Among patients who underwent surgery, curative partial hepatectomy significantly increased from 51 (31.7%) of 161 in 1982–1999 to 110 (89.4%) of 123 in 2011–2021 (p < 0.001). Palliative interventions decreased from 37 (23.0%) in 1982–1999 to 4 (3.3%) in 2011–2021. Liver transplantations also decreased from 28 (17.4%) in 1982–1999 to 3 (2.4%) in 2011–2021; p < 0.001). Over the study period, 16 (1.9%) of 836 patients underwent surgery without antiparasitic treatment (1982–1999: 10/255 (3.9%); 2000–2010: 4/229 (1.7%); 2011–2021: 2/352 (0.6%)) and 59 (7.1%) of 836 were not treated (1982–1999: 36 (14. 1%); 2000–2010: 12 (5.2%), and 2011–2021: 11 (3.1%)).

#### Vital status and survival analysis

In December 2000, 198 (72.3%) of 274 patients diagnosed between 1982 and 2000 were alive, and in December 2021, 493 (78.0%) of 632 patients diagnosed between 2001 and 2021 were alive. More details are presented in Supplementary Table S4.

**Figure 3:**
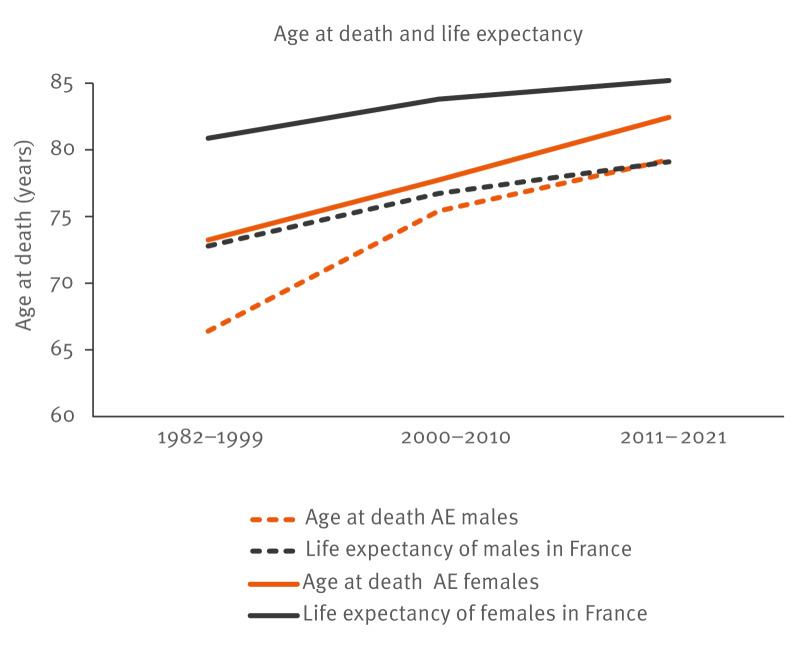
Age at death of patients with alveolar echinococcosis (n = 215) compared with life expectancy of the French population, France, 1982–2021

From the comparison between the age at death of patients with AE and the life expectancy in the French population (INSEE data) for the same periods for males and females, respectively, an average of shortened lifespan of 6.4 and 7.6 years was observed in 1982–1999, and of 0 and 2.8 years in 2011–2021, respectively (Figure 3) and Supplementary Table S5.

AE: alveolar echinococcosis.

Data on life expectancy were obtained from the French National Institute of Statistics and Economic Data (INSEE) (https://www.insee.fr/en/accueil).

**Figure 4:**
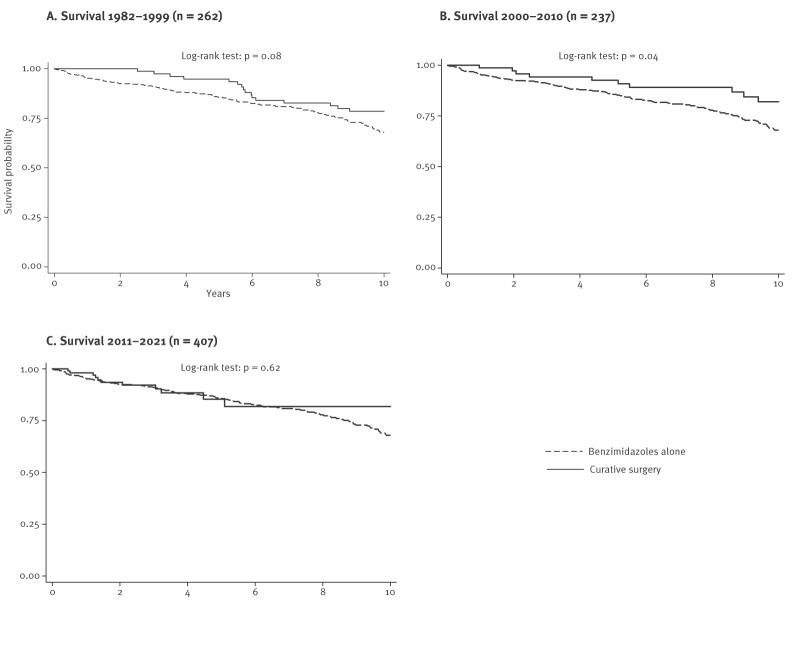
Survival of patients with alveolar echinococcosis, by treatment and follow-up period, France, 1982–2021 (n = 906)

The probability of survival at 10-year follow-up of patients with AE who underwent curative surgery or benzimidazole treatment was compared using Kaplan-Meier analysis (Figure 4). Survival was better after curative surgery than after benzimidazole treatment alone (Figure 4), with a significant difference in 2000–2010 (log-rank test p = 0.04) (Figure 4B). The vital status at 10-year follow-up (all causes of death) was analysed with epidemiological and medical data. In univariate analyses, survival at 10-year follow-up was better for patients diagnosed after 2000 (p = 0.003), those receiving antiparasitic treatment alone or in combination with surgery and curative surgery (p < 0.001), and those treated in a university hospital (p = 0.03). Details are presented in Supplementary Table S6. In contrast, survival was poorer at 10-year follow-up for patients with occupational activity at risk for AE (p = 0.001), and those with weakened immune system (p < 0.001), more details available in Supplementary Table S6. From the multivariable analysis (adjusted for age at AE diagnosis), a diagnosis of AE after 2000, receiving antiparasitic treatment (sub-distribution hazard ratio (SHR) = 0.43; 95% CI: 0.28–0.66; p < 0.001) and living in a suburban area (SHR = 0.48; 95% CI: 0.29–0.78; p = 0.004) (Table 3) were independently associated with a lower risk of all-cause death at 10-year follow-up (SHR = 0.64; 95% CI: 0.48–0.87; p = 0.005). In contrast, male sex (SHR = 1.87; 95% CI: 1.63–2.16; p = 0.004) and living in a DAR (SHR = 1.90; 95% CI: 1.01–3.58; p = 0.048) were associated with a higher risk of all-cause death at 10 years.

AE: alveolar echinococcosis.

Kaplan-Meier survival curves of alveolar echinococcosis patients by treatment with curative surgery and antiparasitic drugs or benzimidazoles alone.

**Table 3:** Factors associated with the cumulative incidence of 10-year all-cause of death of patients with alveolar echinococcosis from a multiple imputation sub-distribution hazard, France, 1982–2021 (n = 906)

Characteristics	Multivariable model		
	SHR	95% CI	p value
Age at AE diagnosis, per increase of 10 years	1.87	1.63–2.16	< 0.001
Male sex	1.53	1.14–2.05	0.004
AE diagnosis after 2000	0.64	0.48–0.87	0.005
Living in DAR 1 or 2	1.90	1.01–3.58	0.048
Living in a suburban area	0.48	0.29–0.78	0.004
AE discovered through screening	0.26	0.07–0.96	0.044
Antiparasitic treatment	0.43	0.28–0.66	< 0.001

AE: alveolar echinococcosis; CI: confidence interval; DAR: area at risk; SHR: sub-distribution hazard ratio.

## Discussion

Based on a national registry, the present study provides an update of epidemiological and clinical data on a rare but severe hepatic parasitic disease in France. The increased number of registered human cases of AE over time could be partially due the increasing visibility of the NRC for Echinococcoses in France. However, the growing prevalence of *E. multilocularis* in foxes since the late 20th century, along with its spread to new areas [5,19], likely explain most of the increase in human AE incidence. Host-related factors, such as immune suppression, and the availability of medical imaging techniques, have also contributed to more AE diagnoses in western Europe in recent years. Despite a limited number of appropriate antiparasitic drugs available, earlier diagnosis and multidisciplinary management of the disease following international recommendations now ensures a far better prognosis for AE patients than was anticipated 40 years ago.

Alveolar echinococcosis is often described as a rural disease, affecting farmers and foresters, especially in regions with cold winters and wide areas of permanent grassland [20,21], such as in eastern and central France [22]. Because of changes in fox habitat, exposure is now more likely to occur in suburban and even urban areas in endemic regions [23,24]. The proportion of farmers diagnosed with AE decreased during the study period, the number of people employed in agriculture has also decreased in France (the national ratio of farmers decreased from 7.1% in 1982 to 1.5% in 2019, based on INSEE data). Recreational activities related to gardening and nature, of people without occupational at-risk activities, has become the main risk factor. Domestic gardens and infected domestic dogs and cats [25] probably contribute to transmission, with studies showing persistence of parasite eggs in the soil [26]. Although the estimated prevalence of the parasite seems to be low in dogs and cats, pets have a close contact with humans, including children. As AE was diagnosed also in children, teenagers and young adults in France, the *E. multilocularis* cycle has been active in France over the last two decades. Family members are at risk, justifying systematic screening of people living close to a recently diagnosed patient. In addition, we observed an increase in AE in people living outside endemic areas, suggesting the spread of AE to the west and north of France, in accordance with the westward spread of *E. multilocularis* in red foxes after 2000 [19]. Establishment of a European registry of AE cases would allow epidemiologists to assess if the trends observed in France are similar in other European countries.

In our study, as in other European studies, the proportion of symptomatic patients at the time of diagnosis has decreased [12,27]. In France, asymptomatic patients represented nearly 60% of the diagnosed cases in 2011–2021. The absence of symptoms cannot be explained by changes in the size or extension of AE lesions in the liver, as no major changes in the PNM scores were observed over time. However, the type of AE lesions at diagnosis seems to have changed. Over the last period, multiple small lesions scattered throughout several liver segments, thus classified as P4 were seen. The P4 lesions in the first period were large, with central necrosis, and usually symptomatic, with severe complications. This raises questions about the validity of the PNM classification to characterise hepatic AE lesions in the 2020s, and suggests that it should be updated to better fit the current situation. The main reason for the significant increase in asymptomatic cases may be the parallel development and availability of medical imaging techniques, which have enabled incidental diagnoses. This observation emphasises the very slow development of *E. multilocularis*-related liver lesions, which are usually seen 5–10 years after becoming infected. The regular use of various medical imaging techniques for the follow-up of patients with malignant and inflammatory diseases is an additional factor [28]. Although previous reports did not indicate a worse prognosis for patients with weakened immune systems [11,27,28], the present study demonstrated a poorer vital status for them. These observations are concerning, especially as chemical and biological immunosuppressive drugs are increasingly used for treatment of diseases previously not curable [11]. This broadens the range of medical specialists that could be confronted with this parasitic disease, and who should be made aware of the possibility of opportunistic AE. In endemic areas, patients can be systematically screened using imaging and serological techniques before long-term immunosuppressive therapy [28]. The teaching and training of radiologists and nuclear medicine specialists should be encouraged, because their expertise is the main asset for early diagnosis.

Retrospective studies have highlighted improved AE prognosis over time [2,29]. Our registry-based analysis confirmed that patient survival has increased over the 40 years of data collection, with a convergence in France of the life expectancy in the general population and age at death of patients suffering from AE. In addition, following the international recommendations for the treatment of patients with AE, that is, treating all patients with ABZ, avoiding palliative operations and favouring curative surgery, was associated with better survival. However, the collected data used for the survival analyses did not include the cause of death, particularly, whether it was directly related to AE. Moreover, in AE patients with weakened immune systems, missing data about the type and the severity of cancer or autoimmune and chronic inflammatory diseases were also an obstacle to proper comparisons with immunosuppressed patients without AE. This was one of the main limitations of our study. Nevertheless, our data showed a benefit of the combination of curative surgery and ABZ, in terms of survival (all causes of death considered). However, it was significant for 2000–2010 only. When the use of palliative surgery decreased after 1999, according to our experience, the patients treated with surgery were more carefully selected, and the number of patients diagnosed with weakened immune systems was still low. This difference was no longer significant in 2011–2021, when blood ABZ-sulfoxide concentration monitoring was easily made available by the NRC-E to French patients and was used to fine-tune the treatment of patients with ABZ alone. These observations suggest that while more effective antiparasitic treatment that could be administered over a shorter period with fewer side effects are needed, ABZ alone is an appropriate alternative for patients who cannot undergo surgery. International recommendations have stressed the importance of a multidisciplinary approach on therapeutic decisions [1,3]. This is supported by the observation of a higher survival of patients living in suburban areas and those treated in university hospitals than for those treated in other healthcare facilities or living in rural DAR. The development of online multidisciplinary consultation meetings should provide more equity in the clinical management of the disease.

## Conclusion

Over the study period of 40 years, we observed an increase in the incidence of AE, the number of asymptomatic cases, cases among patients with weakened immune systems and people living outside of at-risk areas. The survival rate was significantly higher for patients diagnosed after 2000, most likely as a consequence of better treatment of this rare and severe hepatic disease. This study emphasised the importance of registries for neglected or rare diseases. To increase the relevance of this registry, more clinical data should be included (e.g. size of lesions, pharmacological data, follow-up data), and more precise information on the direct or indirect accountability of death to alveolar echinococcosis should be obtained.

## Data Availability

Data can be obtained from the corresponding author upon request.

## Supplementary Data

292000 Supplementary Material
