# Supplementary material for: Epidemiological and clinical characteristics of patients in the alveolar echinococcosis registry, France, 1982 to 2021
Source: Euro Surveill. 2025 Aug 14;30(32):2500041. doi: 10.2807/1560-7917.ES.2025.30.32.2500041 (PMC12355909; doi:10.2807/1560-7917.ES.2025.30.32.2500041)
Supplement: Supplementary Material [file 25-00041_KNAPP_Supplement.pdf]

Supplementary materials

This supplementary material is hosted by Eurosurveillance as supporting information alongside the article “**The French alveolar echinococcosis registry: a 40-year collection of epidemiological and clinical data on 906 patients** “, on behalf of the authors, who remain responsible for the accuracy and appropriateness of the content. The same standards for ethics, copyright, attributions and permissions as for the article apply. Supplements are not edited by Eurosurveillance and the journal is not responsible for the maintenance of any links or email addresses provided therein.

Table of contents

Supplementary Table S1. 2
Supplementary Table S2 3
Supplementary Table S3. 4
Supplementary Table S4. 5
Supplementary Table S5 6
Supplementary Table S6. 7
Supplementary Figure S1. 8
Supplementary Figure S2. 9
Supplementary Figure S3. 10
Supplementary Figure S4. 11
Supplementary Figure S5. 12
Supplementary Methods S1 13
Supplementary Methods S2 13
References 14

Supplementary Table S1.

Epidemiological, clinical, and vital status data at diagnosis documented in the FrancEchino database for French patients with alveolar echinococcosis diagnosed from 1982 to 2021.

Data	Sub-data	Documented item
Epidemiological data	socio-professional data	gender, age
		professional activity at diagnosis
	geographical data	where born and location of residence at diagnosis
	living in the French endemic area or outside	residence in the endemic area. stay (>3 months) in the endemic area
	at risk activity	hunting, contact with foxes, consumption of wild berries/plants, owning a domestic animal, gardening
	professional activity at risk	agriculture activity and other professional at-risk activity (4 categories)
Clinical data	circumstances at the diagnosis	date of diagnosis<br>circumstances of discovery (symptomatic, incidental discovery, or systematic survey), type of symptom (8 items)<br>immunosuppressed patient at diagnosis and type (7 items)
	antiparasitic treatment	treatment type and starting date
	surgical treatment	type (curative, palliative surgery, and liver transplantation (LT)), date, time between diagnosis and surgery/LT
Vital status	vital status of the patient on 12/31/2021	alive/dead/lost to follow-up and date

Supplementary Table S2

Details of PNM scores for French patients with alveolar echinococcosis (2000 to 2021). P: hepatic localization of the parasite, N extra-hepatic involvement of neighboring organs, and M metastasis. Stages I and IIIa: liver only; stage IIIb: liver with or without neighboring organ; stage IV: liver with neighboring organ and/or metastasis.

Stage	PNM	2000-2021	2000-2010	2011-2021
Stages I-IIIa	P1-3N0M0	350 (63.3%)	136 (69.0%)	214 (60.1%)
Stage I	P1N0M0	243 (43.9%)	90 (45.7%)	153 (43.0%)
Stage II	P2N0M0	66 (11.9%)	26 (13.2%)	40 (11.2%)
Stage IIIa	P3N0M0	41 (7.4%)	20 (10.2%)	21 (5.9%)
Stage IIIb	P4N0M0	48 (8.7%)	7 (3.6%)	41 (11.5%)
	P1-3N1M0	49 (8.9%)	22 (11.2%)	27 (7.6%)
	P1N1M0	22 (4.0%)	7 (3.6%)	15 (4.2%)
	P2N1M0	14 (2.5%)	9 (4.6%)	5 (1.4%)
	P3N1M0	13 (2.4%)	6 (3.0%)	7 (2.0%)
Stage IV	P4N1M0	21 (3.8%)	5 (2.5%)	16 (4.5%)
	P1-4N0-1M1	47 (8.5%)	17 (8.6%)	30 (8.4%)
	P1N0M1	10 (1.8%)	5 (2.5%)	5 (1.4%)
	P2N0M1	2 (0.4%)	2 (1.0%)	0 (0%)
	P3N0M1	3 (0.5%)	2 (1.0%)	1 (0.3%)
	P4N0M1	4 (0.7%)	0 (0%)	4 (1.1%)
	P1N1M1	3 (0.5%)	1 (0.5%)	2 (0.6%)
	P2N1M1	1 (0.2%)	1 (0.5%)	0 (0%)
	P3N1M1	4 (0.7%)	2 (1.0%)	2 (0.6%)
	P4N1M1	8 (1.4%)	0 (0%)	8 (2.2%)
	PXN0M1	7 (1.3%)	3 (1.5%)	4 (1.1%)
	PXN1M1	2 (0.4%)	0 (0%)	2 (0.6%)
	P4N1MX	3 (0.5%)	1 (0.5%)	2 (0.6%)
	P0N0-1M0-1	12 (2.2%)	6 (3.0%)	6 (1.4%)
	P0N0M1	10 (1.8%)	4 (2.0%)	6 (1.7%)
	P0N1M0	1 (0.2%)	1 (0.5%)	0 (0.0%)
	P0N1M1	1 (0.2%)	1 (0.5%)	0 (0.0%)
	other PNM	26 (4.7%)	4 (2.0%)	22 (6.2%)
	PXN0M0	14 (2.5%)	0 (0%)	13 (1.8%)
	P1N0MX	2 (0.4%)	2 (1.0%)	0 (0%)
	P2N0MX	1 (0.2%)	0 (0%)	1 (0.3%)
	P4N0MX	1 (0.2%)	0 (0%)	1 (0.3%)
	P1NXMX	2 (0.4%)	0 (0%)	2 (0.6%)
	P1N1MX	1 (0.2%)	0 (0%)	1 (0.3%)
	P2NXMX	1 (0.2%)	0 (0%)	1 (0.3%)
	P4NXMX	2 (0.4%)	0 (0%)	2 (0.6%)
	P2NXM0	1 (0.2%)	0 (0%)	1 (0.3%)
	PXN1M0	1 (0.2%)	1 (0.5%)	0 (0%)
	Total	553	197	356

Supplementary Table S3.

Type and rate of surgical interventions for each period for French patients with alveolar echinococcosis diagnosed in France from 1982 to 2021.

Type of surgery	1982-2021	1982-1999	2000-2010	2011-2021
**Curative partial hepatectomy**	**235 (58.5%)**	**51 (31.7%)**	**74 (62.7%)**	**110 (89.4%)**
Curative partial hepatectomy	231 (57.5%)	51 (31.7%)	72 (61%)	108 (87.8%)
Curative partial hepatectomy and curative surgery on another organ	2 (0.5%)	0	1 (0.8%)	1 (0.8%)
Curative partial hepatectomy and curative tumorectomy	1 (0.2%)	0	0	1 (0.8%)
Curative partial hepatectomy and palliative hepatectomy	1 (0.2%)	0	1 (0.8%)	0
**Palliative partial hepatectomy**	**49 (12.2%)**	**37 (23.0%)**	**8 (6.8%)**	**4 (3.3%)**
**Liver transplant**	**35 (8.7%)**	**28 (17.4%)**	**4 (3.4%)**	**3 (2.4%)**
Liver transplantation alone	17 (4.2%)	13 (8.1%)	2 (1.7%)	2 (1.6%)
Liver transplantation and other interventions	18 (4.5%)	15 (9.3%)	2 (1.7%)	1 (0.8%)
**Other surgery**	**83 (20.6%)**	**45 (28.0%)**	**32 (27.1%)**	**6 (4.9%)**
Curative surgery on another organ	3 (0.7%)	0	2 (1.7%)	1 (0.8%)
Curative tumorectomy	3 (0.7%)	0	1 (0.8%)	2 (1.6%)
Palliative surgery on another organ	9 (2.2%)	1 (0.6%)	6 (5.1%)	2 (1.6%)
Palliative tumorectomy	4 (1.0%)	1 (0.6%)	2 (1.7%)	1 (0.8%)
Internal biliary bypass	24 (6%)	21 (13%)	3 (2.5%)	0
External drainage	2 (0.5%)	2 (1.2%)	0	0
Exploratory laparotomy	38 (9.5%)	20 (12.4%)	18 (15.3%)	0
Total	402	161	118	123

Supplementary Table S4.

Vital status of French alveolar echinococcosis patients as of December 2021 and separately: December 2000 for patients diagnosed from 1982 to 2000 and December 2021 for patients diagnosed from 2001 to 2021.

Vital status in Dec 2021

Yr of first diagnosis	Alive	Last news date <Dec 2021*	Deceased	Years between diagnosis and death	Total
1982-1985	16	11	47	<1 - 37	63
1986-1990	22	19	75	<1 - 31	97
1991-1995	20	15	35	<1 - 28	55
1996-2000	32	25	27	<1 - 23	59
2001-2005	46	38	39	<1 - 19	85
2006-2010	91	63	49	<1 - 14	140
2011-2015	139	86	37	<1 - 10	176
2016-2021	217	71	14	<1 - 4	231
Total	583 (64.3%)	328	323 (35.7%)		906

Vital status in Dec 2000

Year of diagnosis	Alive	Last news date <Dec 2000*	Deceased	Years between diagnosis and death	Total
1982-1985	38	5	25	<1 - 15	63
1986-1990	62	3	35	<1 - 13	97
1991-1995	46	3	9	<1 - 6	55
1996-2000	52	3	7	<1 - 5	59
Total	198 (72.3%)	14	76 (27.7%)		274

Vital status in Dec 2021

Year of diagnosis	Alive	Last news date <Dec 2021*	Deceased	Years between diagnosis and death	Total
2001-2005	46	38	39	<1 – 19	85
2006-2010	91	63	49	<1 – 14	140
2011-2015	139	86	37	<1 – 10	176
2016-2021	217	71	14	<1 – 4	231
Total	493 (78%)	258	139 (22%)		632

*considered alive but lost to follow-up before December 31, 2021.

Supplementary Table S5

Mean age at death in French alveolar echinococcosis patients and life expectancy in the general French population in three period of time. CI, confidence interval.

	Age at death in AE patients [CI 95%]				Life expectancy in general French population	
Period	Female		Male		Female	Male
1982-1999	73.2	[69.2-77.4]	66.4	[61.9-70.9]	80.9	72.8
2000-2010	77.7	[73.9-81.5]	75.4	[72.1-78.9]	83.8	76.7
2011-2021	82.4	[80.1-85.0]	79.2	[76.9-81.6]	85.2	79.1

Supplementary Table S6.

Characteristics of French patients with alveolar echinococcosis and vital status after 10 years of follow-up.

Characteristic	Number of missing values (%)	All patients, N=906	Vital status at 10 years of follow-up		*p*-value*
			Alive*, N=723	Deceased, N=183	
Median age at AE diagnosis (interquartile range), years	0	61 (48-71)	58 (46-68)	72 (60-78)	<0.001
Male gender, % (n/N)	0	51.7% (468/906)	50.1% (362/723)	57.9% (106/183)	0.06
AE diagnosis after 2000, % (n/N)	0	71.2% (645/906)	73.4% (531/723)	62.3% (114/183)	0.003
Living in DAR 1 or 2, % (n/N)	0	85% (770/906)	82.7% (598/723)	94% (172/183)	<0.001
Living in a suburban area, % (n/N)	0	17.3% (157/906)	19.5% (141/723)	8.7% (16/183)	0.001
Patient treated in a University Hospital, % (n/N)	0	83.4% (756/906)	84.8% (613/723)	78.1% (143/183)	0.03
Occupational activity without risk of AE, % (n/N)	166 (18.3%)	31.5% (233/740)	28.9% (174/603)	43.1% (59/137)	0.001
AE discovered through screening, % (n/N)	60 (6.6%)	4% (34/846)	4.7% (32/676)	1.2% (2/170)	0.046
Immune deficiency, % (n/N)	576** (63.6%)	45.8% (151/330)	38.6% (107/277)	83% (44/53)	<0.001
Antiparasitic treatment alone or associated to surgery, % (n/N)	70 (7.7%)	91% (761/836)	93.6% (625/668)	81% (136/168)	<0.001
Curative surgical intervention alone or associated to antiparasitic treatment, % (n/N)	0	25.9% (235/906)	29.2% (211/723)	13.1% (24/183)	<0.001

*Assumed to be alive at least until the latest registered personal contact.

**76.7% of missing data were in periods 1 and 2 (1982-1999 and 2000-2010)

AE: alveolar echinococcosis

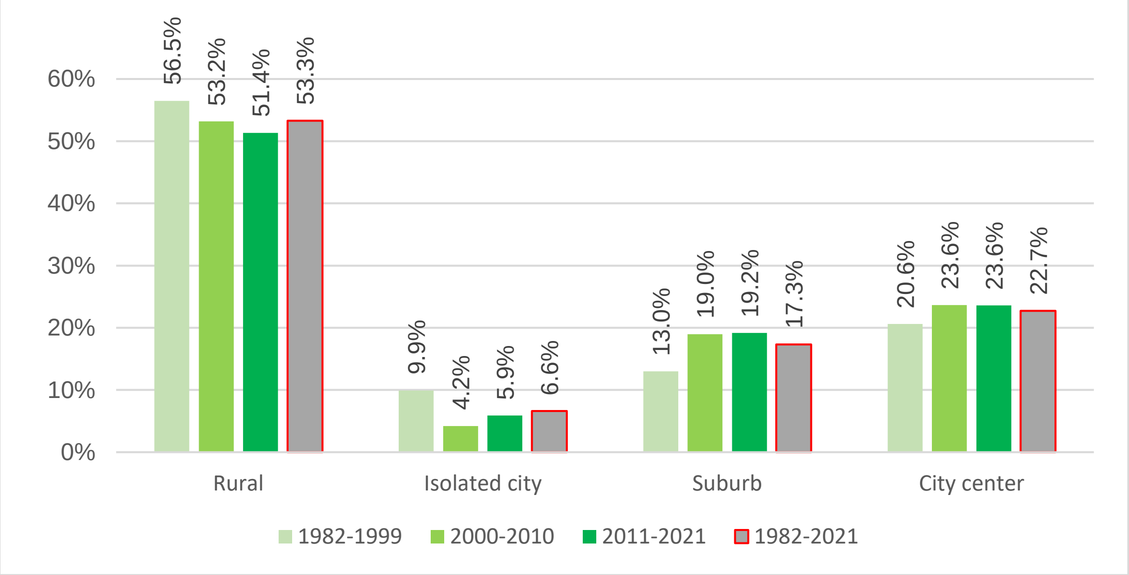

Supplementary Figure S1.

Urban status of the residence of French patients with alveolar echinococcosis at diagnosis over three study periods (from January 1982 to December 2021). Classification of the urbanization status of the French municipalities according to the French National Institute of Statistics and Economic data (INSEE), 2020, https://www.insee.fr/).

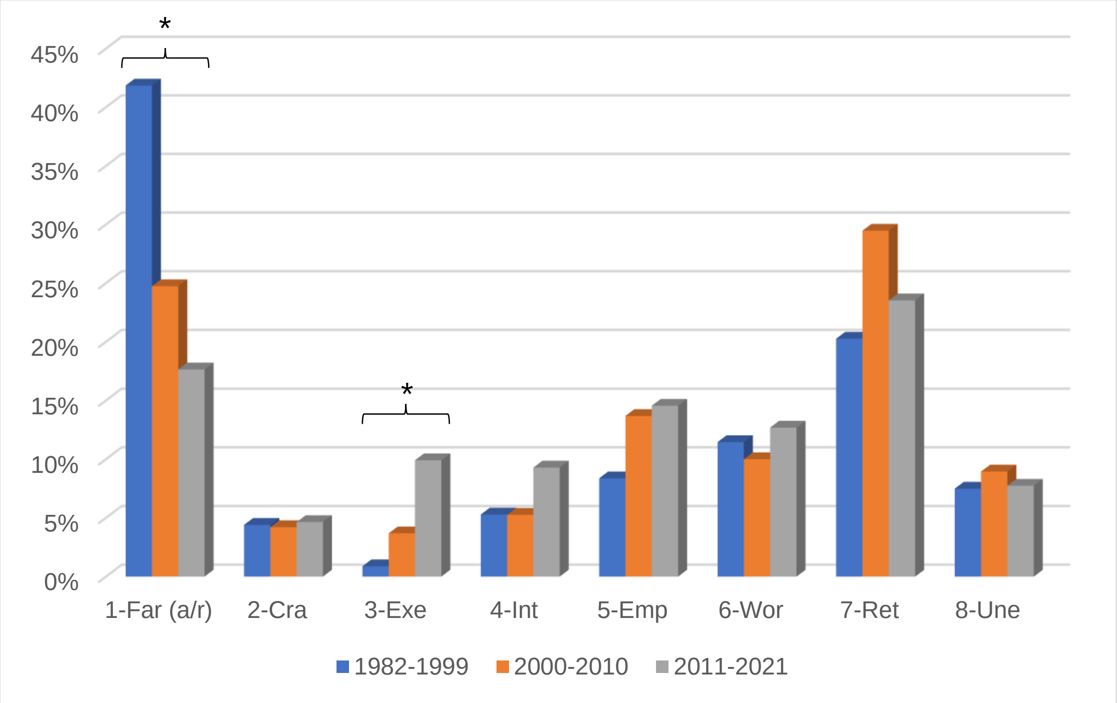

Supplementary Figure S2.

Socio-professional categories (SPC) among the 740 French patients with alveolar echinococcosis diagnosed from January 1982 to December 2021. The SCPs are coded as 1-Far(a/r), farmers (active/retired workers); 2-Cra craftpersons, shopkeepers; 3-Exe, executives and intellectual professionals; 4-Int, intermediary professions; 5-Emp, employees; 6-Wor, workers; 7-Ret, retired (non-farmers), and 8-Une, unemployed/students. *significant differences among the periods considered.

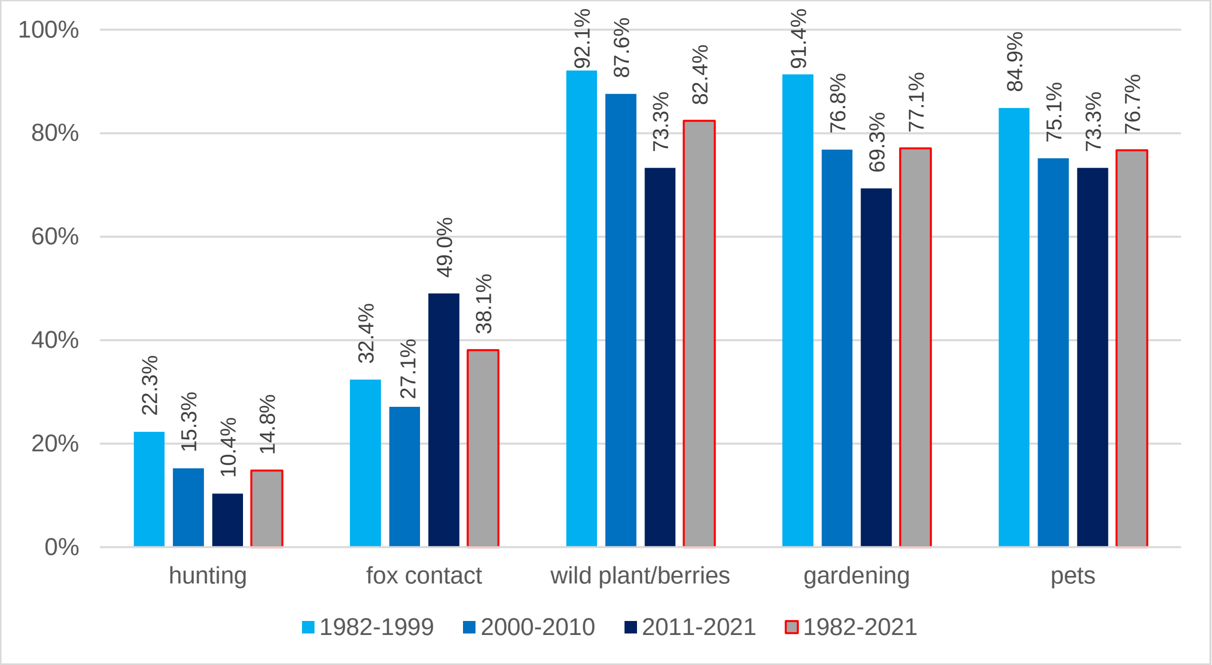

Supplementary Figure S3.

Recreational at-risk activities for *Echinococcus multilocularis* exposure declared by 567 French patients with alveolar echinococcosis from 1982 to 2021.

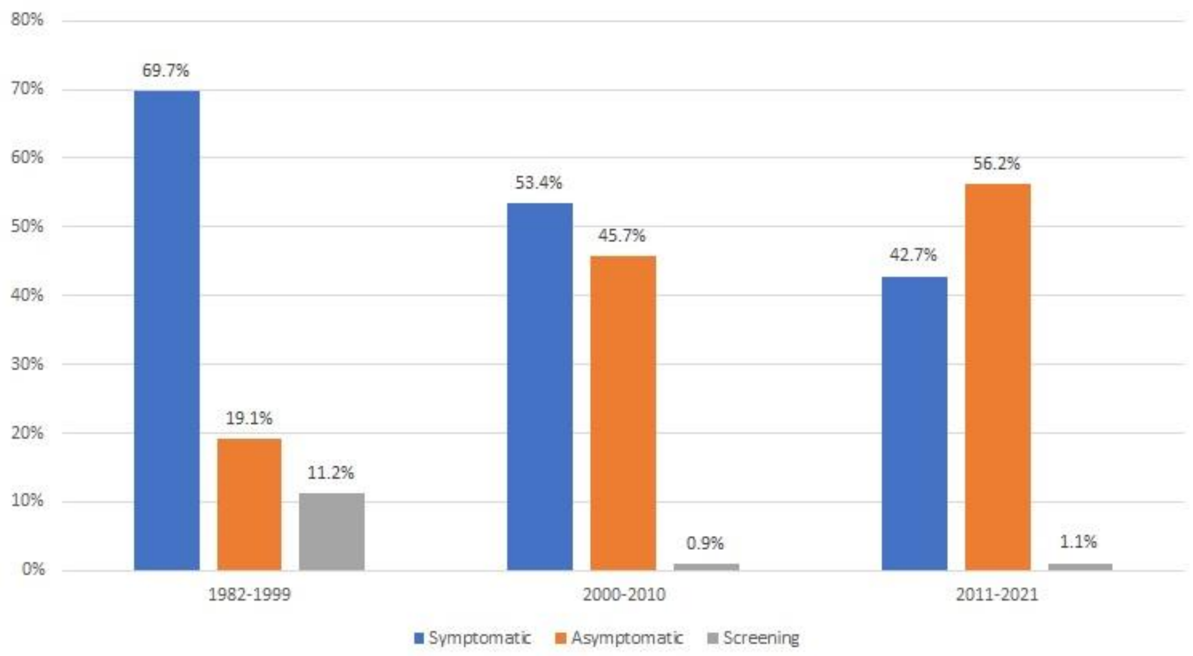

Supplementary Figure S4.

Circumstances of diagnosis for 846 French patients with a diagnosis of alveolar echinococcosis per period considered from 1982 to 2021.

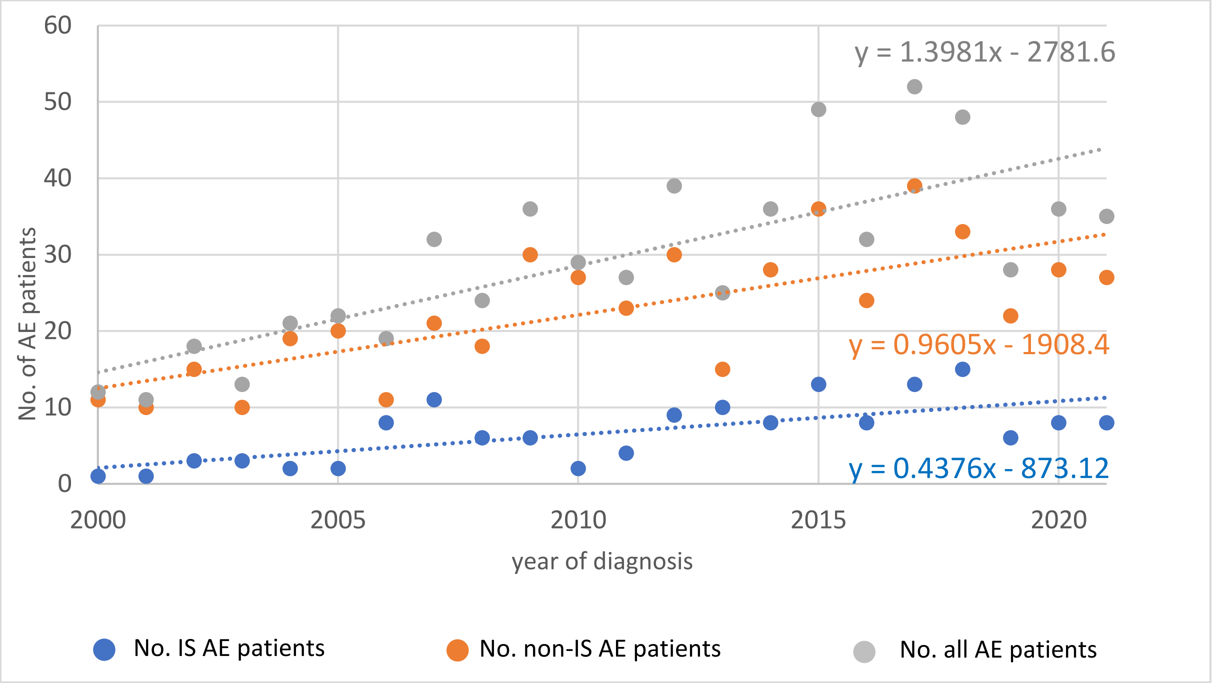

Supplementary Figure S5.

Annual incidence of alveolar echinococcosis (AE) diagnosis from 2000 to 2021 for all French AE patients (all), patients with immunosuppression (IS), and patients without IS (non-IS).

Supplementary Methods S1

- *Location of residence*

The urbanization status of French municipalities (according to the 2020 data from the French National Institute of Statistics and Economic (INSEE) data, https://www.insee.fr/) and the location of the patients' residence at diagnosis were combined. Four categories were recorded: city centers (C), suburbs (B), isolated cities (assimilated in unique urban units) (I), and rural cities (R). In 2007, urban areas represented 20.5% of all municipalities (C+B+I) in France, and included 77.5% of the French population [1].

The geographical location of the patients' residence at the French administrative division "*Département*" scale for the three periods considered were mapped using Philcarto (http://philcarto.free.fr) and the geographical information system Quantum GIS 3.16 (QGIS Development Team, 2020. QGIS Geographic Information System (http://www.qgis.org).

- *Recreational activities*

Five at-risk recreational activities practiced by patients before the alveolar echinococcosis diagnosis were recorded, as highlighted in the literature for *Echinococcus multilocularis* exposure [2–4]: (1) hunting, (2) contact (direct or indirect) with red foxes, (3) consumption of raw wild berries, plants, or both, (4) gardening, and (5) owning a dog, cat, or both.

- *Occupational activity*

The occupational activity at diagnosis for the patients with AE was recorded and classified among eight socio-professional categories (SPC) according to the INSEE (SPC 1: Far(a/r), farmers (active/retired workers); SPC 2: Cra, craftpersons, shopkeepers; SPC 3: Exe, executives and intellectual professionals; SPC 4: Int, intermediary professions; SPC 5: Emp, employees; SPC 6: Wor, workers; SPC 7: Ret, retired (non-farmers), and SPC 8: Une, unemployed/students). Potentially occupational at-risk activities (OARA) for *E. multilocularis* exposure, as highlighted in the literature [2,4], were classified into four categories based on their being outdoor activities leading to direct contact with soil or animals: OARA-1: farmers (active or retired), shepherds, gardeners, market gardeners, OARA-2: farm workers, OARA-3: forestry workers, and OARA-4: other at-risk outdoor occupations.

Supplementary Methods S2

- *Survival data and survival probability*

The characteristics of AE patients and their vital status were compared after 10 years of follow-up. Data are reported as medians (interquartile ranges) for continuous variables and percentages for categorical variables. Continuous data were compared using Welch's t-test and categorical data using Pearson's chi-square test or Fisher's exact test, as appropriate.

The competing-risks regression model was constructed with 10-year all-cause death as the interest event and the loss to follow-up as the competing event. A Fine-Gray sub-distribution hazard (risk-based)

model was constructed to identify covariates independently associated with the cumulative incidence of the event of interest over time. To handle missing data, the multiple imputation method was implemented using the substantive model compatible fully conditional specification (SMC-FCS) of Bartlett et al. [5]. Multiple imputation was used to create and analyze 75 multiply imputed datasets. The final multiple imputation multivariable model was fitted using a backward elimination process as the covariate selection procedure.

References

1. Clanché F, Rascol O. Le découpage en unités urbaines en 2010. Insee Première [Internet]. 2011 Aug;(1364). Available from: http://www.insee.fr

2. Conraths FJ, Probst C, Possenti A, Boufana B, Saulle R, La Torre G, et al. Potential risk factors associated with human alveolar echinococcosis: Systematic review and meta-analysis. PLoS Negl Trop Dis. 2017 Jul;11(7):e0005801.

3. Kreidl P, Allerberger F, Judmaier G, Auer H, Aspöck H, Hall AJ. Domestic pets as risk factors for alveolar hydatid disease in Austria. Am J Epidemiol. 1998 May 15;147(10):978–81.

4. Stefaniak M, Derda M, Zmora P, Nowak SP. Risk Factors and the Character of Clinical Course of the *Echinococcus multilocularis* Infection in Patients in Poland. Pathogens. 2023 Jan 28;12(2):199.

5. Bartlett JW, Seaman SR, White IR, Carpenter JR, Alzheimer's Disease Neuroimaging Initiative*. Multiple imputation of covariates by fully conditional specification: Accommodating the substantive model. Stat Methods Med Res. 2015 Aug;24(4):462–87.
